# Genistein Ameliorates Ischemia/Reperfusion-Induced Renal Injury in a SIRT1-Dependent Manner

**DOI:** 10.3390/nu9040403

**Published:** 2017-04-20

**Authors:** Wei-Fang Li, Kang Yang, Ping Zhu, Hong-Qian Zhao, Yin-Hong Song, Kuan-Can Liu, Wei-Feng Huang

**Affiliations:** 1 Medical College, China Three Gorges University, Yichang 443002, China; lwf199023@163.com (W.-F.L.); yangkang0218@126.com (K.Y.); 15271506793@163.com (H.-Q.Z.); syh728@126.com (Y.-H.S.); 2 Department of Medicine, the First College of Clinical Medical Science, China Three Gorges University, Yichang 443002, China; topgan2000@163.com; 3 Institute for Laboratory Medicine, Fuzhou General Hospital, PLA, Fuzhou 350025, China; 4 Department of Medicine, Columbia University Medical Center, New York, NY 10032, USA; 5 Dongfang Hospital, Xiamen University, Fuzhou 350025, China

**Keywords:** genistein, SIRT1, renal ischemia-reperfusion

## Abstract

Renal ischemia/reperfusion (I/R) injury continues to be a complicated situation in clinical practice. Genistein, the main isoflavone found in soy products, is known to possess a wide spectrum of biochemical and pharmacological activities. However, the protective effect of genistein on renal I/R injury has not been well investigated. In the current study, we explore whether genistein exhibits its renal-protective effects through SIRT1 (Sirtuin 1) in I/R-induced mice model. We found the treatment of genistein significantly reduced renal I/R-induced cell death, simultaneously stimulating renal cell proliferation. Meanwhile, SIRT1 expression was up-regulated following the administration of genistein in renal region. Furthermore, pharmacological inhibition or shRNA-mediated depletion of SIRT1 significantly reversed the protective effect of genistein on renal dysfunction, cellular damage, apoptosis, and proliferation following I/R injury, suggesting an indispensible role of the increased SIRT1 expression and activity in this process. Meanwhile, the reduced p53 and p21 expression and increased PCNA (Proliferating Cell Nuclear Antigen) expression were blocked after the depletion of SIRT1 compared with the genistein treatment group in the renal I/R process. Hence, our results provided further experimental basis for the potential use of genistein for the treatment of kidney disease with deficiency of SIRT1 activity.

## 1. Introduction

Renal I/R injury is a major cause of acute kidney injury (AKI), which is a clinical condition of frequent occurrence and high mortality [1,2]. I/R injury can increasingly develop into AKI in many clinical settings, such as kidney transplantation, renal artery angioplasty, sepsis, and partial nephrectomy, or by the action of vasoconstrictor drugs and certain hypotensive states [3]. There are many factors that likely contribute to I/R-induced renal injury. However, the exact molecular mechanisms underlying renal I/R injury are not completely understood. Various pharmaceuticals have been identified for the treatment of renal I/R injury in the laboratory, such as AICAR (5-amino-4-imidazolecarboxamide riboside-1-b-D-ribofuranoside), yohimbine, and pioglitazone [4,5,6]. However, these are limited, and few renal protectants have been successfully translated into clinical applications. Therefore, it is critical and urgent to develop safe and effective drugs for treating IR-induced renal injury. 

SIRT1, an NAD^+^ (nicotinamide adenosine denucleotide)-dependent protein deacetylase, exerts cytoprotective effects through multiple mechanisms, such as anti-apoptosis, anti-oxidative, and anti-inflammation effects and the regulation of mitochondrial biogenesis and autophagy [7]. Many studies have shown that SIRT1 can regulate multiple physiological processes, including gene transcription, glucose homeostasis, cellular stress response, and immune response through its capability of deacetylating various factors. The target proteins of deacetylation by SIRT1 include histones, transcriptional regulators (p53; forkhead box O transcription factors, FoxOs; Nuclear factor κB, NF-κB; hypoxia-inducible factors 2α, hypoxia-inducible factors 2α, HIF 2α), enzymes (acetyl-CoA synthase1, AceCS1), and other signaling molecules such as peroxisome proliferator activated receptor γ co-activator 1α (PGC1-α), thus affecting crucial cellular pathways in physiological and pathological processes [8]. The renal protected effects of SIRT1 have been demonstrated in various kidney diseases [9]. For example, SIRT1 preserves podocyte function by tuning claudin-1, which is a key regulator of albuminuria and glomerular function [10]. He et al. [11] have reported that SIRT1 protects the kidney medulla from oxidative stress-induced cellular injury by regulating the induction of COX2 (cytochrome c oxidase subunit II). In addition, in mesangial cells, SIRT1 can prevent oxidative stress-induced apoptosis by the deacetylation of p53 [12]. SIRT1 inhibits TGF β1 (transforming growth factor β1)-induced apoptosis in glomerular mesangial cells via Smad7 deacetylation [13]. Recent studies have suggested that SIRT1 protects against I/R-induced renal injury by attenuating apoptosis and promoting regeneration [14]. The protection caused by SIRT1 is closely involved in the inhibition of the p53 signaling pathway. Specific deletion of p53 in the proximal tubule cells protects kidneys from I/R-induced renal functional and histologic deterioration [15,16]. Moreover, caloric restriction has been shown to provide protection against I/R-induced renal injury [17]. SIRT1 expression is strongly influenced by calorie restriction. While multiple genes respond to caloric restriction, SIRT1 is at least thought to be involved in the health benefits attributed to long-term caloric restriction [7].

Genistein (4′,5,7-trihydroxyisoflavone), the most extensively studied soy isoflavone thus far, is a polyphenolic non-steroidal compound commonly used as a dietary supplement. Because genistein possesses oestrogen-like biological activity, its biological effects have been explored in conditions such as cancer, inflammation, and apoptosis [18]. Previously, we also found that genistein inhibited cancer stem cell-like properties and reduced the chemoresistance of gastric cancer [19]. Genistein has been reported to protect against I/R-induced cerebral injury in the rat [20], and it has a protective role on I/R-induced small intestine injury [21]. Additionally, Canyilmaz, E. et al. demonstrated that the administration of genistein protects mice against radiation-induced nephrotoxicity [22]. It is of particular relevance that the physiological activity of genistein as a phytoestrogen is closely related to SIRT1 activity [23]. Genistein has multiple beneficial properties, including low toxicity, it has potential in clinical treatment. However, the role and potential mechanism of genistein in I/R-induced kidney injury remains unknown.

In this study, we aimed to determine the role of genistein in I/R-induced kidney injury and then dissect the exact mechanism responsible for the treatment of I/R-induced kidney injury. Thus, it will be helpful for us to establish guidance for the treatment of I/R-induced kidney injury.

## 2. Materials and Methods 

### 2.1. Experimental Animals, I/R, Genistein Treatment, and SIRT1 Inhibition

Seven- to nine-week-old BALB/c mice were supplied by the China Three Gorges University Laboratory Animal Center, and they were housed in an air-conditioned room with 12-h light and dark cycles. All of the experiments were carried out in accordance with NIH Guidelines for the Care and Use of Laboratory Animals. The animals were kept in sterile cages (maximum of five per cage) and fed standard rodent chow and allowed free access to water ad libitum. All of the experimental protocols were approved by our School of Medicine Animal Care and Use Committee. An animal’s ability to drink water, feed, ambulate, and its general appearance were evaluated after operation. They were monitored three times in 24 h after genistein treatment. 

An established model of the renal I/R-induced injury was established previously [24]. Briefly, an abdominal midline incision was made, and right nephrectomy was performed under anesthesia. Mice were anaesthetized using 100 mg/kg ketamine and 0.75 mg/kg chlorpromazine intraperitoneally. Left renal ischemia was induced by clamping renal pedicles for 45 min with microvascular clamps and then subjected to reperfusion. Mice in the sham group underwent right nephrectomy and were treated as indicated in the specific experiment. The mice were hydrated with warm saline during the operation, and the body temperature was maintained constantly at 37 °C using a heating pad until awake. The wounds were sutured after removal of the clips, and the animals were allowed to recover.

Genistein (Sigma Aldrich, St. Louis, MO, USA; dissolved in 0.9% sodium chloride containing 1% dimethyl sulphoxide) was given (intravenous injection, i.v.) 30 min before the induction of ischaemia. For the pharmacological inhibition of SIRT1 activity, mice were treated (i.v.) with Sirtinol (Sigma Aldrich, St. Louis, MO, USA; 1 mg/kg dissolved in 0.9% sodium chloride containing 1% dimethyl sulfoxide) 60 min before the induction of ischemia. The others received the same volume of vehicle. For the inhibition of SIRT1 by lentivirus, a 31-gauge needle was used to inject 100 μL of ultracentrifugation-purified lentivirus (lentivirus, LV; LV-control or LV-shSIRT1, ≈3 × 10^7^ TU) at the lower pole of the left kidneys parallel to the long axis and was slowly removed 72 h later [25]; then, induction of ischemia was performed.

For the dose-dependent analyses of genistein effects on I/R-induced renal injury, mice were randomized into five groups (*n* = 6/group): (1) sham group; (2) I/R group; (3) G5 group; (4) G10 group; and (5) G15 group. In the sham group, mice received the same volume of vehicle without I/R; in the I/R group, mice received the same volume of vehicle with I/R; in the G5 group, mice were pre-treated with 5 mg/kg genistein following I/R; in the G10 group, mice were pre-treated with 10 mg/kg genistein following I/R; in the G15 group, mice were treated with 15 mg/kg genistein. The mice were decapitated after 24 h of reperfusion, and blood samples were collected for the analysis of biochemical measurements. The kidneys were excised and then washed with ice-cold saline for further analysis.

For the time-dependent analyses of the genistein effects on I/R renal-induced injury, mice were randomized into three groups (*n* = 24/group): (1) sham group; (2) I/R group; and (3) G15 group. In the sham group, mice received the same volume of vehicle without I/R; in the G15 group, mice were pre-treated with 15 mg/kg genistein following I/R. Six mice in each group were decapitated at 12, 24, 48, and 72 h of the reperfusion period, and blood samples were collected for the analysis of biochemical measurements. The kidneys were excised and then washed with ice-cold saline for further analysis.

For the inhibition of SIRT1 by Sirtinol, mice were randomized into four groups (*n* = 6/group): (1) vehicle group; (2) Sirtinol group; (3) G15 group; and (4) Sirtinol + G15 group. Ischemia was induced in all groups. In the vehicle group, mice received the same volume of vehicle; in the Sirtinol group, mice were pre-treated with 1 mg/kg Sirtinol; in the G15 group, mice were pre-treated with 15 mg/kg genistein; in the Sirtinol + G15 group, mice were treated with 15 mg/kg genistein and 1 mg/kg Sirtinol. Sirtinol and genistein were administered as described above. The mice were decapitated after 24 h of reperfusion and blood samples were collected for the analysis of biochemical measurements. The kidneys were excised and then washed with ice-cold saline for the further analysis.

For the inhibition of SIRT1 by lentivirus carrying shRNA targeting SIRT1, mice were randomized into four groups (*n* = 6/group): (1) LV-control group; (2) LV-shSIRT1 group; (3) G15 group; and (4) LV-shSIRT1 + G15 group. Ischemia was induced in all of the groups. In the LV-control group, mice were injected with lentivirus carrying scrambled shRNA and were pre-treated with the same volume of vehicle; in the LV-shSIRT1 group, mice were injected with lentivirus carrying SIRT1 shRNA; in the G15 group, mice were injected with lentivirus carrying scrambled shRNA and were pre-treated with 15 mg/kg genistein; in the G15 + LV-shSIRT1 group, mice were injected with lentivirus carrying SIRT1 shRNA and were pre-treated with 15 mg/kg genistein. Lentivirus injection and genistein treatment were performed as described above. The mice were decapitated at 24 h of the reperfusion period, and trunk blood samples were collected for the analysis of biochemical measurements. The kidneys were excised and then were washed with ice-cold saline for further analysis.

### 2.2. Biochemical Determinations

Serum was obtained from blood samples to measure blood urea nitrogen (BUN) and serum creatinine (Scr) using an automatic biochemistry analyzer (Hitachi 7060, Tokyo, Japan) after reperfusion.

### 2.3. Kidney Histology

For histological examination, the kidneys were fixed in 4% paraformaldehyde, embedded in paraffin, sectioned at 4-μm thickness, and stained with hematoxylin and eosin (H&E). Tubular injury was scored on a scale of 0–4 based on the percentage of tubules with necrosis, dilatation, cast formation and cell lysis: 0, no damage; 1, 5%–25%; 2, 25%–50%; 3, 50%–75%; and 4, >75%. The morphological changes from the cross-sectional area cortex and outer medulla were evaluated by a pathologist according to the acute tubular necrosis (ATN) scoring system in a blinded manner [26].

### 2.4. Immunohistochemistry 

For immunohistochemical (IHC) studies, 4-μm-thick sections were microwaved in 0.01 mol/L sodium citrate (pH 6.0) three times for 5 min each. The primary antibodies used were anti-SIRT1 (1:100; Abcam, Cambridge, UK), anti-cleaved caspase-3 (Cell Signaling Technology, Danvers, MA, USA) and anti-PCNA (1:200; Santa Cruz, Dallas, TX, USA), respectively. IHC was performed using the UltrasensitiveTM SP (Mouse/Rabbit) IHC kit (Mai-xin Biotechnology Co., Fuzhou, China). Images were acquired using a digital camera (Olympus, Tokyo, Japan) at 400× magnification, and the results were analyzed by Image-Pro Plus 6.0 (Media Cybernetics, Rockville, MD, USA). The results were defined as the number of positive cells/total number of cells.

### 2.5. TUNEL Assay

Apoptosis detection was performed using the terminal deoxynucleotidyl transferase-mediated digoxigenindeoxyuridine nick-end labelling (TUNEL) assay (Roche Diagnostics, Mannheim, Germany) according to the protocols of the manufacturer. Briefly, samples were incubated in equilibration buffer for 5 min, followed by incubation in the labelling reaction reagent for 1 h at 37 °C, and DNA fragments in apoptotic cells were recognized. The positive cellular counts of staining were assessed at 400× magnification using five randomly selected fields, and the apoptosis index was expressed as the percentage of positive cells in high-power fields. Data were averaged.

### 2.6. Western Blotting

Proteins from kidney samples were isolated using lysis buffer followed by centrifugation. Western blot analysis of the proteins in mouse kidneys was performed according to standard protocols. Immunoblotting was performed using anti-SIRT1 antibody (1:5000; Abcam, Cambridge, UK), anti-cleaved caspase-3 antibody (1:1000; Cell Signaling Technology, Danvers, MA, USA), anti-pro-caspase-3 antibody (1:1000; Zen BioScience, Chengdu, China), anti-PCNA antibody (1:2000; Santa Cruz, Dallas, TX, USA), anti-p53 antibody (1:1000; Santa Cruz, Dallas, TX, USA), anti-acetyl-p53 antibody (K381) (1:1000; Abcam, Cambridge, UK), and anti-p21 antibody (1:2000; Santa Cruz, Dallas, TX, USA). The blots were detected using the Immobilon Western Chemiluminescent HRP Substrate kit (Millipore, Danvers, MA, USA) followed by exposure to Kodak-X-Omat film (Shanghai, China).

### 2.7. Statistical Analysis 

All of the statistical analyses were performed using GraphPad Prism 5.0 software. The values are expressed as the mean ± SEM (standard error of the mean). Pair-wise comparisons were performed using Student’s *t*-test (two-tailed), and multiple-group comparisons were performed using one-way ANOVA with Bonferroni’s post-hoc test. A *p*-value < 0.05 was considered significant.

## 3. Results

### 3.1. Genistein Protects the Kidney Against I/R Injury

**Figure 1:**
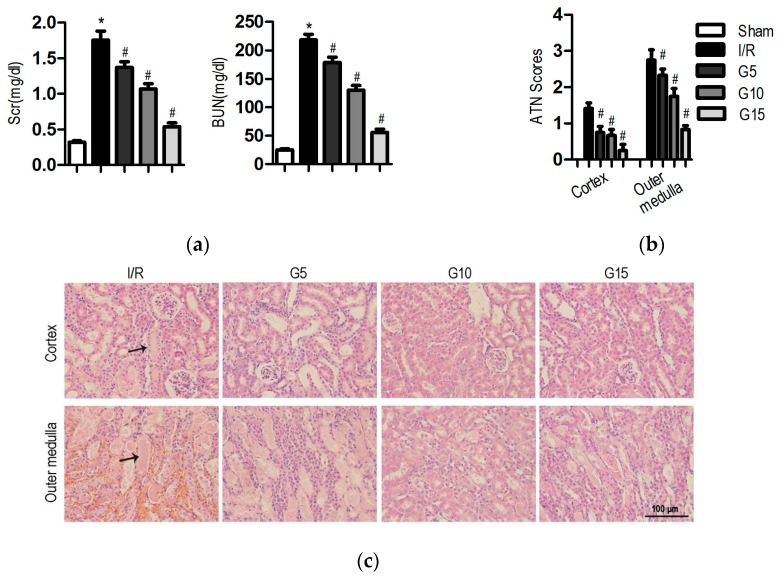
Effect of genistein on kidney injury following I/R injury. In the sham group, mice received the same volume of vehicle without I/R; in the I/R group, mice received the same volume of vehicle with I/R; in the G5 group, mice were pre-treated with 5 mg/kg genistein following I/R; in the G10 group, mice were pre-treated with 10 mg/kg genistein following I/R; and in the G15 group, mice were treated with 15 mg/kg genistein following I/R. The mice were decapitated after 24 h of reperfusion for further analysis. The data were expressed as the mean ± SEM; *n* = 6; * *p* < 0.05 vs. Sham; ^#^ *p* < 0.05 vs. I/R. (**a**) The Scr levels and BUN levels were examined 24 h after surgery; (**b**) A semi-quantitative assessment of the lesion was performed by a pathologist in a blinded manner according to the ATN–scoring system. Each tubular segment visible in the cortex and the outer medulla was evaluated; (**c**) Representative images of the cortex and outer medulla in different groups stained with H&E. Original magnification, 400×. The black arrows indicate the areas of I/R-induced tissue damages.

Renal I/R injury increased the Scr (serum creatinine) and BUN (blood urea nitrogen) levels to 1.8 ± 0.1 and 218.4 ± 9.7 mg/dL, respectively, from the levels in the sham group (0.3 ± 0.02 and 25.3 ± 1.7 mg/dL, respectively; Figure 1a, *p* < 0.05). The genistein-pretreated group showed significant reduction in the elevated Scr and BUN levels in a dose-dependent manner. Genistein at the dose of 15 mg/kg significantly decreased the Scr (0.5 ± 0.05 mg/dL, *p* < 0.05) levels and BUN (56.1 ± 5.4 mg/dL, *p* < 0.05) levels compared with those of the I/R group.

To investigate the effect of genistein on I/R-induced renal tubular damage, kidney sections were stained with H&E. Tubular dilatation, necrosis, brush border loss, and cast formation were evident in the I/R group. However, after pre-treatment with genistein, the damage was limited to mild swelling of the tubular epithelial cells, and less histological damage was observed with the H&E stain (Figure 1c). Pretreatment with genistein significantly reduced the tubular necrosis score in both the cortex and medulla compared with the score in sections from mice with I/R injury (Figure 1b).

**Figure A1:**
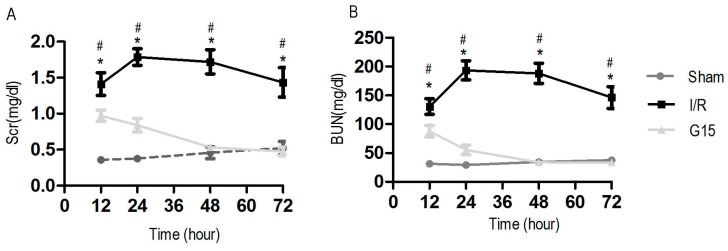
Effect of genistein on renal function at different time points. In the sham group, mice received the same volume of vehicle without I/R; in the I/R group, mice received the same volume of vehicle with I/R; in the G15 group, mice were treated with 15 mg/kg genistein. The mice were decapitated after 12, 24, 48, and 72 h of reperfusion for renal functional analysis. The data were expressed as the mean ± SEM; *n* = 6; * *p* < 0.05 vs. Sham; ^#^ *p* < 0.05 vs. I/R. The Scr (**A**) levels and BUN (**B**) levels were examined 12, 24, 48, and 72 h after reperfusion.

**Figure A2:**
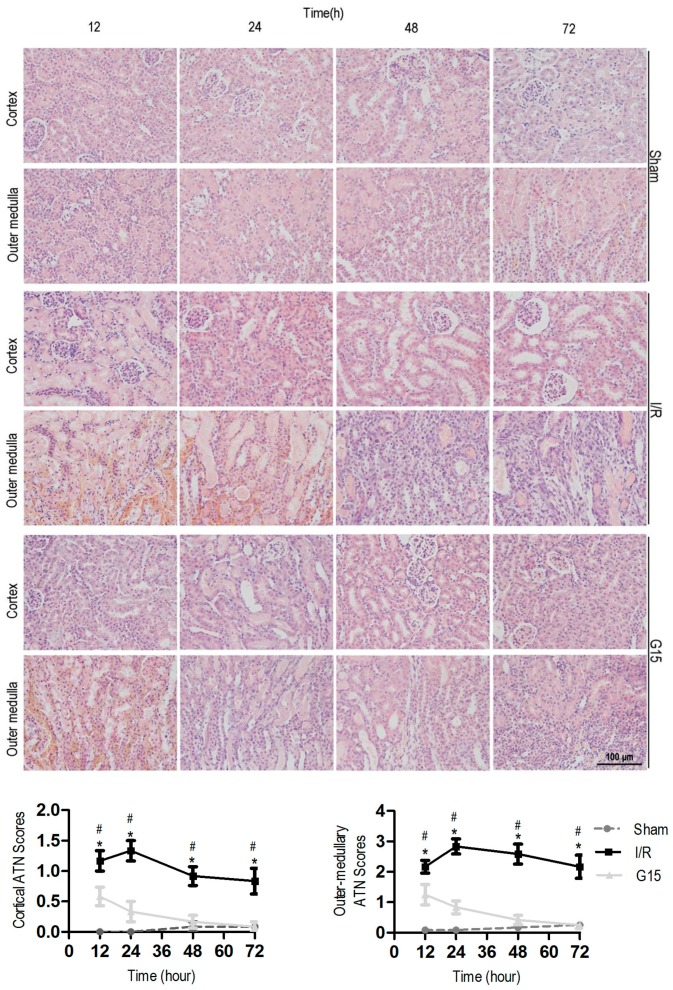
Effect of genistein on renal histology in I/R-induced injury at different time points. Mice were treated as described in supplemental Figure A1. The data were expressed as the mean ± SEM; *n* = 6; * *p* < 0.05 vs. Sham; ^#^ *p* < 0.05 vs. I/R. (**Top**) Representative images of the cortex and outer medulla at 12, 24, 48, and 72 h of the reperfusion period stained with H&E. Original magnification, 400×; (**Bottom**) Semi-quantitative assessment of the lesion was performed by a pathologist in a blinded manner according to the ATN–scoring system. Each tubular segment visible in the cortex and outer medulla was evaluated.

To further explore the protective effects of genistein on I/R-induced renal injury, mice were treated with 15 mg/kg genistein or vehicle 30 min before the induction of ischemia, followed by 12, 24, 48, and 72 h of reperfusion. Genistein treatment led to a significant decrease in the elevated Scr and BUN levels in the I/R group of mice at 12, 24, 48, and 72 h post I/R injury (Figure A1). Genistein treatment at 48 h post I/R injury had returned the Scr and BUN levels to those of the sham group. Examination of the cortical and outer-medullary regions of the kidneys confirmed the results of the aforementioned functional studies (Figure A2).

### 3.2. Genistein Increases SIRT1 Expression in Renal Cells after Renal I/R-Induced Injury

**Figure 2:**
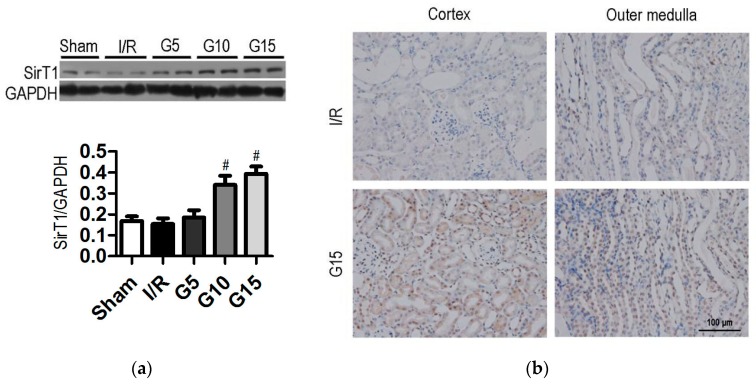
Effect of genistein on SIRT1 expression following I/R injury. Mice were treated as described in Figure 1. The data were expressed as the mean ± SEM; *n* = 6; ^#^ *p* < 0.05 vs. I/R. (**a**) Western blots analysis for SIRT1 expression in mouse kidney tissues. Densitometric analysis of the SIRT1 expression by Image-Pro Plus 6.0. GAPDH was calibrated; (**b**) Representative images of SIRT1 expression in the kidney, as determined by IHC. Original magnification, 400×. The black arrows indicate the positive cells.

**Figure A3:**
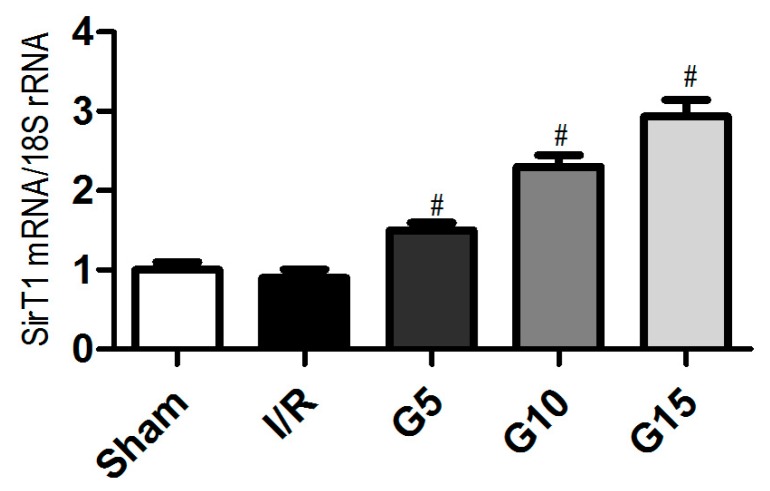
Effect of genistein on SIRT1 mRNA expression following I/R injury. In the sham group, mice received the same volume of vehicle without I/R; in the I/R group, mice received the same volume of vehicle with I/R; in the G5 group, mice were pre-treated with 5 mg/kg genistein following I/R; in the G10 group, mice were pre-treated with 10 mg/kg genistein following I/R; in the G15 group, mice were treated with 15 mg/kg genistein. The mice were decapitated at 24 h of the reperfusion period for further analysis. The data were expressed as the mean ± SEM; *n* = 6; * *p* < 0.05 vs. Sham; ^#^ *p* < 0.05 vs. I/R. The SIRT1 mRNA levels for specific genes were normalized to 18S rRNA levels.

Previous studies have demonstrated that the regulation of SIRT1 by naturally occurring dietary polyphenols is beneficial in the therapeutic intervention of various diseases [27]. To determine whether SIRT1 is also involved in the protective effect of genistein, we analyzed the protein and mRNA expression in renal tissues. No significant differences occurred in SIRT1 expression between the I/R group and the sham group (Figure 2a and Figure A3). Interestingly, pre-treatment with 10 and 15 mg/kg genistein dramatically increased the expression of SIRT1 protein and mRNA (Figure 2a and Figure A3, *p* < 0.05). The mRNA and protein levels of SIRT1 in the 15 mg/kg genistein-treated group were 2.3- and 1.6-fold higher than those in the I/R group (*p* < 0.05), respectively. The upregulation of SIRT1 protein was further confirmed by the IHC assay (Figure 2b). The results showed that pre-treatment with 15 mg/kg genistein significantly increased SIRT1 expression in the renal cortex and outer medulla compared with that in the I/R group. These data indicate that SIRT1 may contribute to the protective effects of genistein on tubular damage and the loss of renal function in I/R injury.

### 3.3. Genistein Inhibits Apoptosis and Increases Proliferation after Renal I/R-Induced Injury

**Figure 3:**
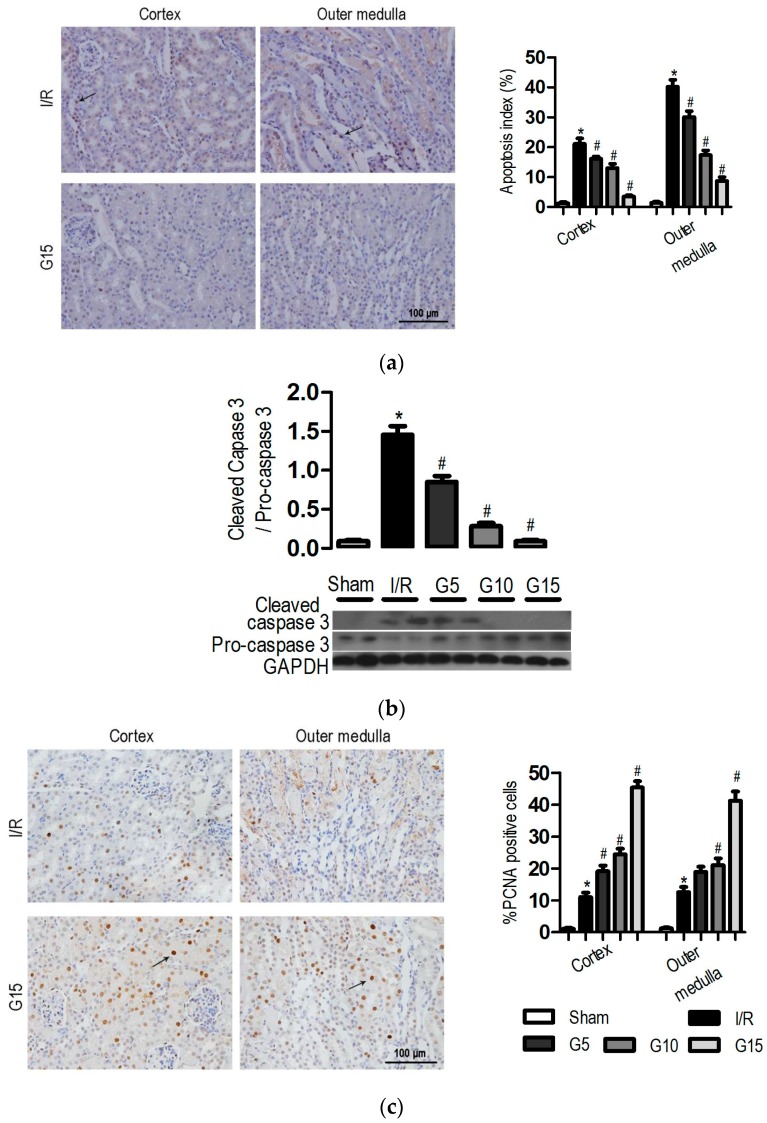
Effect of genistein on the cellular apoptosis and proliferation following I/R injury. Mice were treated as described in Figure 1. The data were expressed as the mean ± SEM; *n* = 6; * *p* < 0.05 vs. Sham; ^#^ *p* < 0.05 vs. I/R. (**a**) Representative photomicrograph of TUNEL-positive cells stained kidney tissues from the I/R and G15 groups and the percentage of positive cells is illustrated; (**b**) Cleaved caspase-3 and pro-caspase-3 expression was analyzed by western blotting; (**c**) Representative photomicrograph of PCNA-positive cells stained kidney tissues from the I/R and G15 groups and the percentage of positive cells is illustrated. Original magnification, 400×. The black arrows indicate the positive cells.

Several lines of experimental evidence have supported a crucial role for apoptosis in the pathogenesis of I/R-induced injury [28]. Apoptotic cells were detected in the kidney of all groups using terminal deoxynucleotidyl transferase-mediated dUTP nick-end labelling (TUNEL) staining. As shown in Figure 3a, mice with I/R injury had more TUNEL-positive cells predominantly in the tubules of the outer medulla than in the cortex region. Pre-treatment with genistein (5, 10, and 15 mg/kg) resulted in a significant decrease in the number of apoptotic cells (30.1% ± 2.0%, 17.3% ± 1.6%, and 8.7% ± 1.3%, respectively) compared with that in the I/R group (40.2% ± 2.3%) in the outer medulla regions (Figure 3a, *p* < 0.05). Meanwhile, the level of cleaved caspase-3 in the kidney treated with genistein (5, 10, and 15 mg/kg) was significantly lower than that in the I/R group (Figure 3b, *p* < 0.05). This finding is consistent with the TUNEL assay.

Renal cell proliferation was assessed using proliferating cell nuclear antigen (PCNA). Few PCNA-positive proliferating cells were detected in the kidneys of sham-operated mice. The number of PCNA-positive cells was significantly increased in the renal cortex (11.1% ± 1.3%) and outer medulla (12.6% ± 1.6%) following I/R (Figure 3c, *p* < 0.05). Pre-treatment with genistein (5, 10, and 15 mg/kg) resulted in a significant increase of the number of PCNA-positive cells in the kidney. The number of PCNA-positive cells for the 15 mg/kg genistein-treated group was significantly higher (45.4% ± 2.0% and 41.3% ± 2.8% for cortex and outer medulla) than that in I/R mice (*p* < 0.05). These results suggested that genistein inhibits apoptosis and promotes proliferation and then prevents the mice from I/R-induced renal injury.

### 3.4. The SIRT1 Inhibitor Abolishes the Protective Effects of Genistein on I/R-Induced Injury

**Figure 4:**
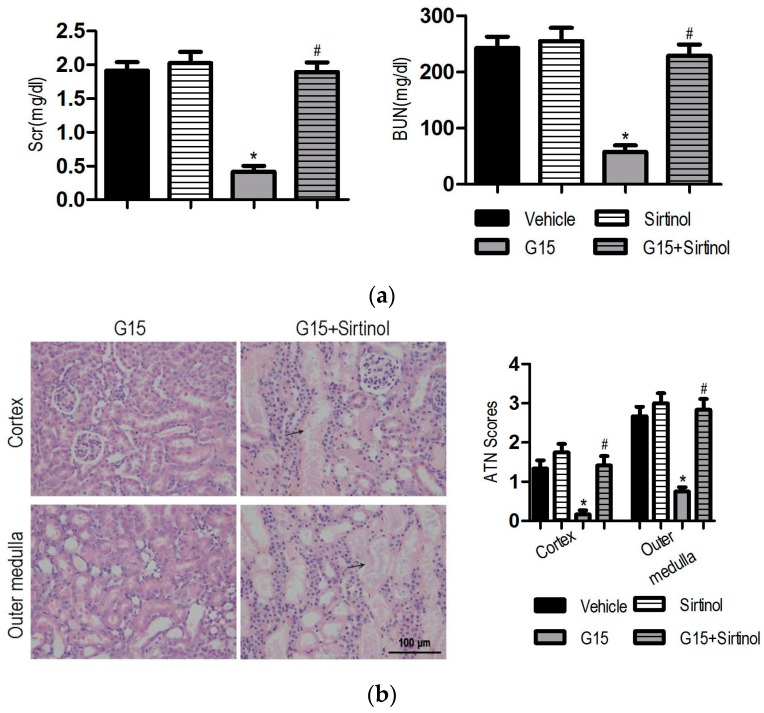
Sirtinol abolishes the protective effect of genistein on renal function and histology in I/R-induced injury. In the vehicle group, mice received the same volume of vehicle; in the Sirtinol group, mice were pre-treated with 1 mg/kg Sirtinol; in the G15 group, mice were pre-treated with 15 mg/kg genistein; in the Sirtinol + G15 group, mice were treated with 15 mg/kg genistein and 1 mg/kg Sirtinol. Ischemia was induced in all of the groups. The mice were decapitated at 24 h of the reperfusion period for further analysis. The data were expressed as the mean ± SEM; *n* = 6; * *p* < 0.05 vs. Sham; ^#^ *p* < 0.05 vs. I/R. The Scr (**a**, **left**) levels and BUN (**a**, **right**) levels were examined 24 h after reperfusion. (**b**, **left**) Representative images of the cortex and outer medulla from the G15 and G15 + Sirtinol groups stained with H&E. Original magnification, 400×. The black arrows indicate the areas of I/R-induced tissue damages. (**b**, **right**) A semi-quantitative assessment of the lesion was performed by a pathologist in a blinded manner according to the ATN–scoring system. Each tubular segment visible in the cortex and outer medulla was evaluated.

To investigate whether SIRT1 is required for the renal-protective effects of genistein against I/R-induced injury, Sirtinol, a SIRT1 inhibitor, was administered 60 min before the induction of ischemia with or without genistein. Sirtinol preconditioning slightly aggravated I/R-induced renal injury. The Scr, BUN, and injury score in Sirtinol-treated mice exhibited higher levels than those of the vehicle mice, although no significant difference occurred between these two groups (Figure 4). Notably, Sirtinol abrogated the protective effect of genistein. The reduction of Scr and BUN in genistein-treated mice was inhibited by Sirtinol. Consistent with this result, the kidney of G15 + Sirtinol mice displayed severe and extensive injury, with widespread loss of the brush border, tubular dilation, and vacuolization (Figure 4). 

**Figure 5:**
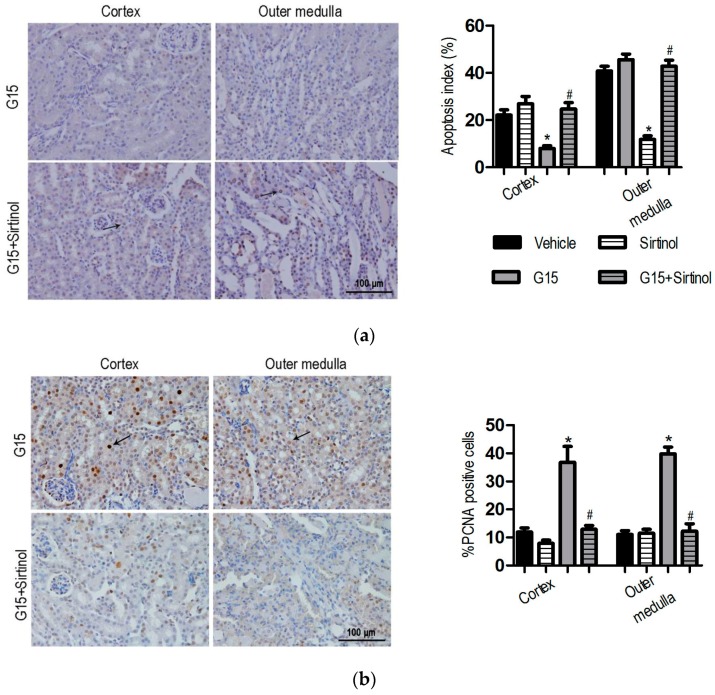
Mice were treated as described in Figure 4. The data were expressed as the mean ± SEM; *n* = 6; * *p* < 0.05 vs. Vehicle; ^#^ *p* < 0.05 vs. G15. (**a**) Representative photomicrograph of TUNEL-positive from the G15 and G15 + Sirtinol groups and the percentage of positive cells were illustrated; (**b**) Representative photomicrograph of PCNA-positive from the G15 and G15 + Sirtinol groups and the percentage of positive cells were illustrated. Original magnification, 400×. The black arrows indicate the positive cells.

**Figure A4:**
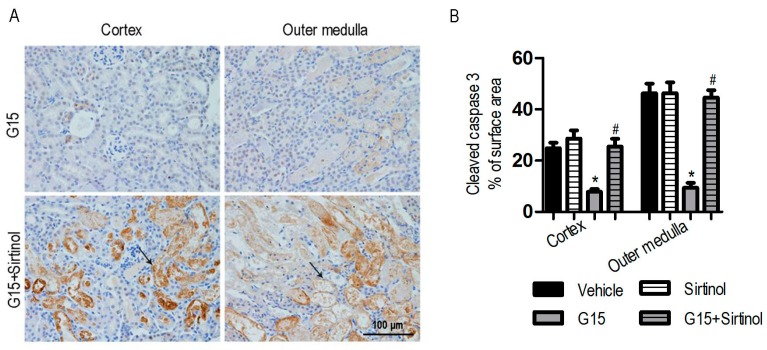
Sirtinol reverses the effect of genistein on apoptosis in I/R-induced acute renal injury. Mice were treated as described in supplemental Figure 4. The data were expressed as the mean ± SEM; *n* = 6; * *p* < 0.05 vs. Vehicle; ^#^ *p* < 0.05 vs. G15. (**A**) Representative photomicrograph of cleaved caspase-3-stained kidney tissues from the G15 and G15 + Sirtinol groups. Original magnification, 400×; (**B**) Cleaved caspase-3-positive cells were counted, and the percentage of positive cells is illustrated. The black arrows indicate positive cells.

**Figure A5:**
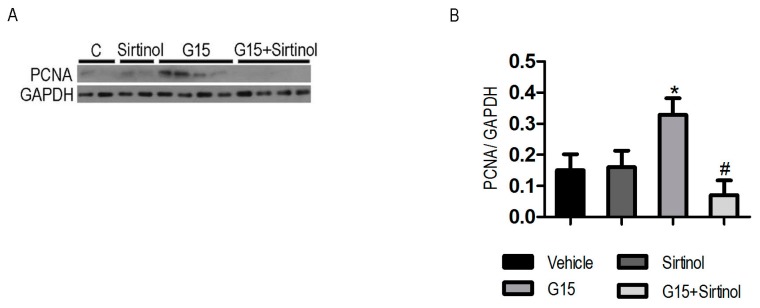
Sirtinol abrogates the effect of genistein on PCNA expression in I/R-induced acute renal injury. Mice were treated as described in supplemental Figure 4. The data were expressed as the mean ± SEM; *n* = 6; * *p* < 0.05 vs. Vehicle; ^#^ *p* < 0.05 vs. G15. (**A**) Representative western blots for PCNA expression in mice kidney tissues; (**B**) Densitometric analysis of the PCNA expression by Image-Pro Plus 6.0. GAPDH was calibrated.

Similarly, tubular cell apoptosis was also determined by the TUNEL assay and analysis of cleaved caspase-3 expression. No significant difference occurred in the apoptosis and proliferation of tubular cells between Sirtinol-treated mice and I/R mice (Figure 5 and Figure A4). Pre-treatment with Sirtinol abolished the protective effect of genistein on I/R-induced apoptosis (Figure 5a). In addition, the promoted effect of genistein on renal proliferation was abrogated by Sirtinol following I/R (Figure 5b). Lower renal PCNA expression following I/R was also observed in Sirtinol-treated mice using immunoblotting (Figure A5), suggesting that the protective effects of genistein depend on SIRT1 activation in the kidney with I/R injury.

### 3.5. SIRT1 Depletion Eliminates the Protective Effects of Genistein on I/R-Induced Injury

**Figure 6:**
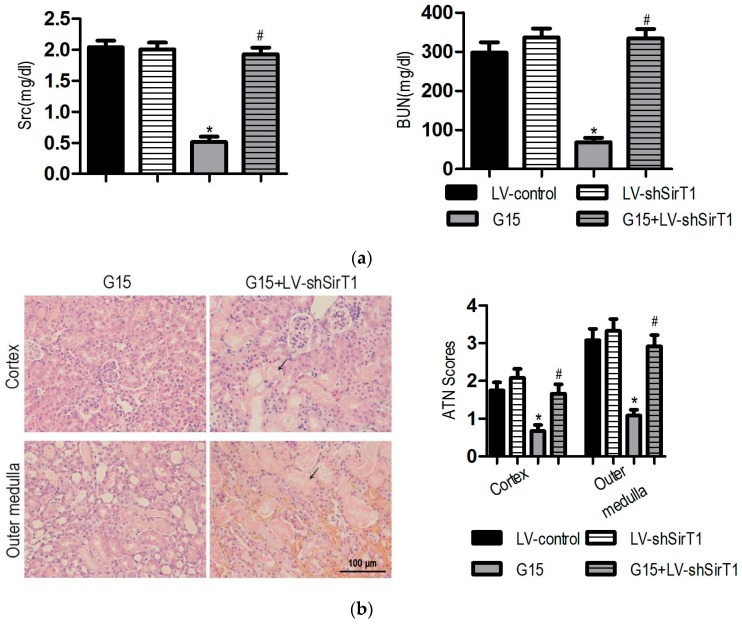
SIRT1 depletion abrogates the protective effect of genistein on renal function and histology in I/R-induced injury. In the LV-control, mice were injected with lentivirus carrying scrambled shRNA and pre-treated with the same volume of vehicle; in the LV-shSIRT1 group, mice were injected with lentivirus carrying SIRT1 shRNA; in the G15 group, mice were injected with lentivirus carrying scrambled shRNA and were pre-treated with 15 mg/kg genistein; in the LV-shSIRT1 + G15 group, mice were injected with lentivirus carrying SIRT1 shRNA and were pre-treated with 15 mg/kg genistein. Ischemia was induced in all of the groups. The mice were decapitated after 24 h of reperfusion for further analysis. The data were expressed as the mean ± SEM; *n* = 6; * *p* < 0.05 vs. Sham; ^#^ *p* < 0.05 vs. I/R. The Scr (**a**, **left**) levels and BUN (**a**, **right**) levels were examined 24 h after reperfusion. (**b**, **left**) Representative images of the cortex and outer medulla from G15 and G15 + LV-shSIRT1 groups stained with H&E. Original magnification, 400×. The black arrows indicate the areas of I/R-induced tissue damages. (**b**, **right**) Semi-quantitative assessment of the lesion was performed by a pathologist in a blinded manner according to the ATN-scoring system. Each tubular segment visible in the cortex and outer medulla was evaluated.

**Figure A6:**
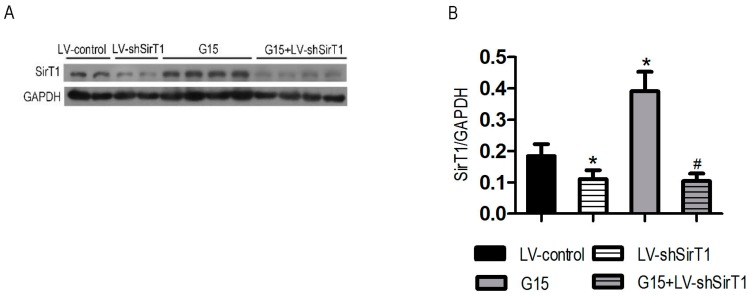
Western blot analyses demonstrating the knock-down of SIRT1 in kidney tissues. Seventy-two hours later after the injection of LV-control or LV-shSIRT1, genistein, or the same volume of vehicle. For the LV-control group and G15 groups, mice were pre-treated with vehicle; for the LV-shSIRT1 group, mice were pre-treated with vehicle; for the G15 and LV-shSIRT1 + G15 groups, mice were pre-treated with 15 mg/kg genistein. Ischemia was induced in all of the groups. The mice were decapitated after 24 h of reperfusion for the western blot analysis. The data were expressed as the mean ± SEM; *n* = 6; * *p* < 0.05 vs. Sham; ^#^ *p* < 0.05 vs. I/R. (**A**) Representative western blots for SIRT1 expression in mouse kidney tissues; (**B**) Densitometric analysis of the SIRT1 expression by Image-Pro Plus 6.0. GAPDH was calibrated.

**Figure A7:**
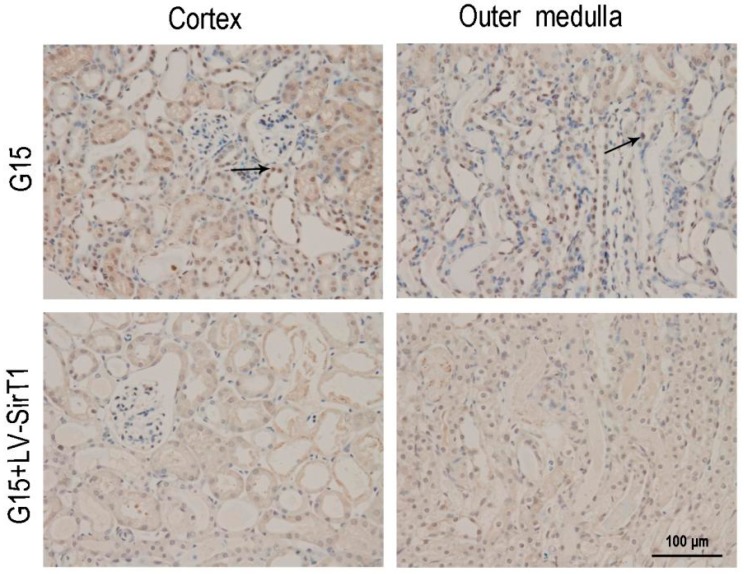
IHC analyses demonstrating the knock-down of SIRT1 in kidney tissues. Mice were treated as described in supplemental Figure 7. Representative photomicrograph of SIRT1-stained kidney tissues from the G15 and G15 + LV-shSIRT1 groups. Original magnification, 400×. The black arrows indicate positive cells.

**Figure A8:**
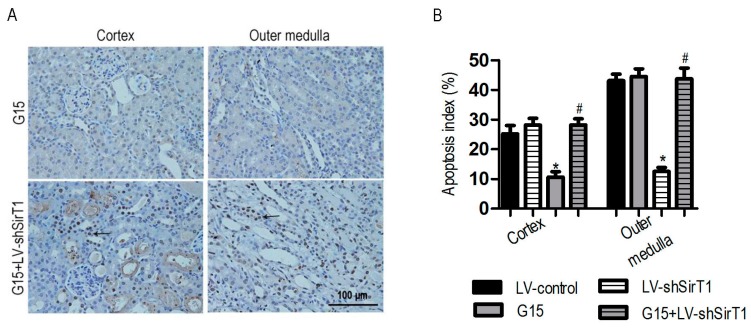
SIRT1 depletion reverses the effect of genistein on apoptosis in I/R-induced acute renal injury. Mice were treated as described in supplemental Figure 7. The data were expressed as the mean ± SEM; *n* = 6; * *p* < 0.05 vs. Sham; ^#^ *p* < 0.05 vs. I/R. (**A**) Representative photomicrograph of TUNEL-stained kidney tissues from the G15 and G15 + Sirtinol groups. Original magnification, 400×; (**B**) TUNEL-positive cells were counted and the percentage of positive cells is illustrated. The black arrows indicate positive cells.

**Figure A9:**
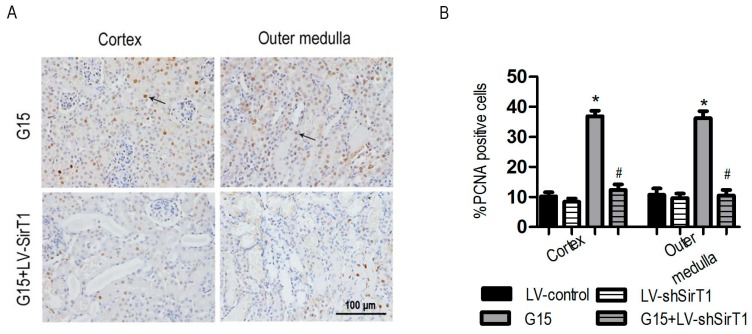
SIRT1 depletion reverses the increased effect of genistein on cellular proliferation in I/R-induced acute renal injury. Mice were treated as described in supplemental Figure 7. The data were expressed as the mean ± SEM; *n* = 6; * *p* < 0.05 vs. Sham; ^#^ *p* < 0.05 vs. I/R. (**A**) Representative photomicrograph of PCNA-stained kidney tissues from the G15 and G15 + Sirtinol groups. Original magnification, 400×; (**B**) PCNA-positive cells were counted, and the percentage of positive cells is illustrated. The black arrows indicate positive cells.

Because both SIRT1 and SIRT2 are inhibited by Sirtinol, the specific contribution of SIRT1 to the protective effects of genistein on I/R-induced injury was determined using shRNA-mediated knockdown with lentivirus. SIRT1 protein was significantly induced by genistein in kidneys injected with lentivirus carrying the scrambled shRNA cassette; however, its expression was significantly lower in I/R- and genistein-treated mice receiving lentivirus with the SIRT1 shRNA cassette (Figure A6). Knockdown of SIRT1 was verified by IHC analysis (Figure A7). Consistent with the result using the SIRT1 inhibitor Sirtinol, knockdown of SIRT1 with shRNA significantly reversed the effect of genistein on renal dysfunction (Figure 6a), cellular damage (Figure 6b), apoptosis (Figure A8), and proliferation (Figure A9) following I/R injury. Thus, the protective effects of genistein depend on SIRT1 expression in the kidney with I/R injury.

### 3.6. The Protective Effects of Genistein Is Associated with the SIRT1/p53 Axis

**Figure 7:**
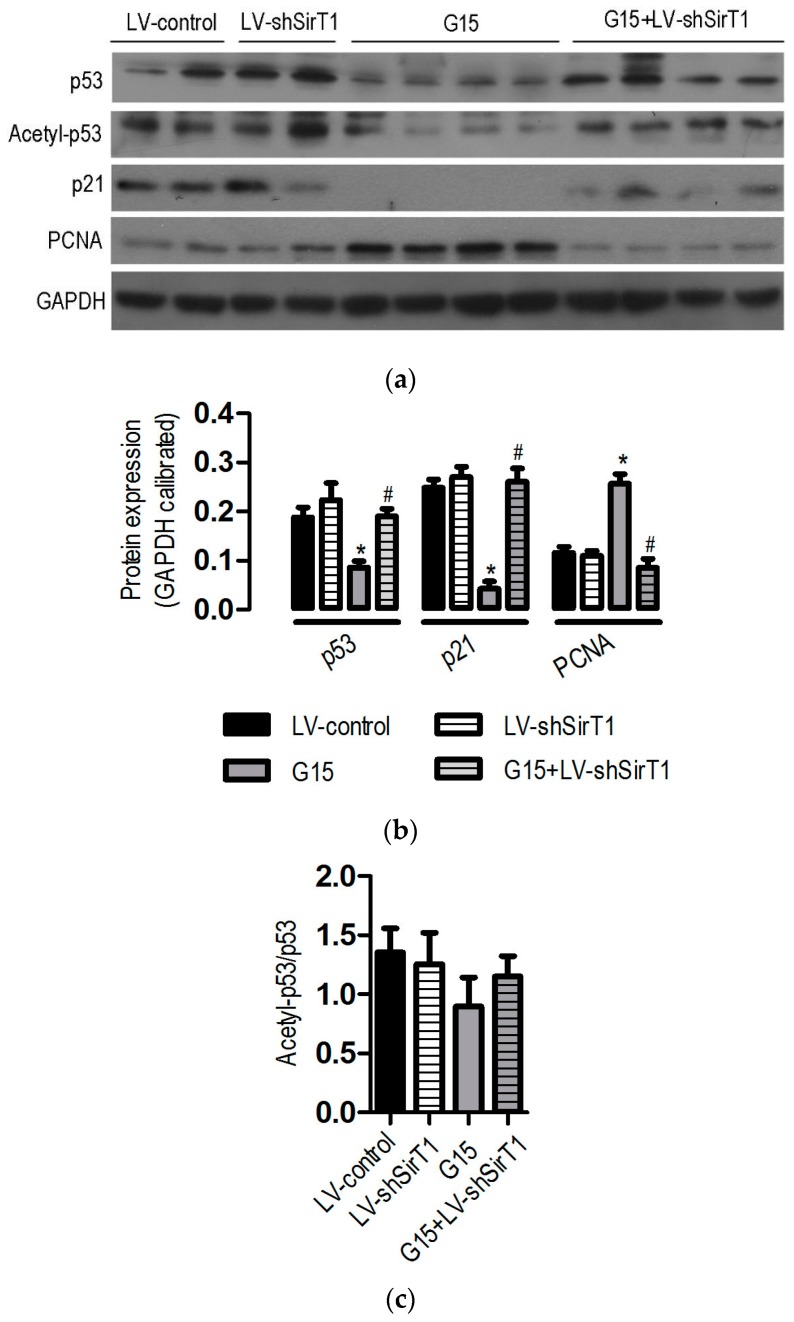
Effect of SIRT1 depletion on protein expression following I/R-induced renal injury. Mice were treated as described in Figure 6. The data were expressed as the mean ± SEM; *n* = 6; * *p* < 0.05 vs. LV-control; ^#^ *p* < 0.05 vs. G15. (**a**) Representative western blots for p53, Acetyl-p53, p21, and PCNA expression in mouse kidney tissues; (**b**) Densitometric analysis of p53, p21, and PCNA expression by Image-Pro Plus 6.0. GAPDH was calibrated; (**c**) Densitometric analysis of Acetyl-p53 expression by Image-Pro Plus 6.0. Total p53 was calibrated.

It has been reported that the expression of p53 is regulated by SIRT1, which then exerts its anti-apoptotic and proliferative effect, thereby contributing to the pathogenesis of ischemic renal injury [14]. Of interest is to investigate whether SIRT1/p53 axis contributes to the protective effects of genistein on I/R-induced injury [15,16]. Our western blot analyses revealed that SIRT1/p53 signaling was also involved in the protective effects of genistein: (1) genistein reduced the expression of p53 and; (2) no significant difference occurred in p53 between LV-shSIRT1-treated mice and LV-control mice; (3) by contrast, the reduction of p53 caused by genistein was significantly prevented by SIRT1 depletion; (4) in addition, the level of the cell cycle-related p21, a target of p53, was significantly inhibited in the kidneys of genistein-treated mice; (5) knock-down of SIRT1 with the shRNA cassette largely blocked the inhibitory effect of genistein on p21 expression; (6) there were inverse correlations between the expression of p53 and p21 and PCNA in the kidneys of different groups following I/R (Figure 7a,b). However, it did not significantly alter the acetylation status of p53 (Figure 7c). Taken together, all of these data strongly suggested that SIRT1/p53 is important for the protective effects of genistein on I/R-induced injury.

## 4. Discussion

Renal ischemia-reperfusion is a common cause of acute kidney injury (AKI), which is a common clinical complication characterized by an abrupt decrease in the glomerular filtration rate. Despite the improvement in therapeutic methods, including renal replacement therapy, the overall mortality of AKI is estimated to be 50% [2]. There are several pathologic processes contributing to AKI, including endothelial and epithelial cell death, intratubular obstruction, and changes in the local microvascular blood flow, as well as immunological and inflammatory processes. Therefore, the mechanisms underlying I/R damage to kidneys are most likely multifactorial and interdependent, involving hypoxia, inflammatory responses, and free radical damage [1]. Effective treatments for I/R-induced renal injury in clinics are still absent. It is crucial to screen effective drugs to treat I/R-induced renal injury. It is well established that the inhibition of apoptotic signalling and cell death is an effective measure to relieve I/R in a murine model. Pre-ischaemic activation of the A1 AR protects against I/R injury in vivo through mechanisms that reduce necrosis and apoptosis [29], and other studies have suggested that tubular cell regeneration and proliferation play an important role in the recovery of AKI [14,28]. As a plant-derived isoflavone, genistein is a polyphenolic non-steroidal compound, and its biological activity has been widely explored in cancer, inflammation, and apoptosis. Recently, it has been reported that genistein plays a neuro-protective role in ischemic insults [30]. As a member of the Sirtuin family, SIRT1 belongs to the typical class III histone deacetylases (HDAC). SIRT1 has been involved in influencing a wide range of cellular processes including aging, transcriptional reprogramming, apoptosis, inflammation, and stress resistance, as well as energy efficiency and alertness during low-calorie situations [7,31]. Moreover, it can also control circadian clocks and mitochondrial biogenesis, suggesting that SIRT1 is an important age-related protective factor against I/R-induced kidney injury. Activation of SIRT1 attenuated I/R-induced renal injury; conversely, the ablation of one allele of the SIRT1 gene significantly resulted in higher susceptibility to I/R-induced kidney injury. Furthermore, the kidney-specific overexpression of SIRT1 could also protect against cisplatin-induced AKI [32]. In this study, renal function and morphology were significantly recovered 24 h after renal I/R injury following pretreatment with genistein (5, 10, and 15 mg/kg). Meanwhile, renal function and morphology were markedly recovered at 12, 24, 48, and 72 h after renal I/R injury following pretreatment with 15 mg/kg genistein. These results strongly support that genistein is an effective agent for the treatment of I/R-induced kidney injury. Consistent with our results, anti-apoptosis activity caused by genistein is also the important mechanism for its neuro-protective role in ischemic insults [30]. In addition, we also showed that the protective roles of genistein in I/R-induced renal injury are associated with a higher cellular proliferation rate, which was evaluated by the PCNA level. All of these studies indicated that genistein and SIRT1 both have multiple roles in many physiological processes, suggesting that they may also contribute to the repair of renal ischemia/reperfusion (I/R) injury.

Previously, many studies have revealed the close relationship between genistein and SIRT1, and the expression of SIRT1 and its activity were stimulated by isoflavones such as resveratrol and quercetin [27]. However, the effect of genistein on SIRT1 expression and activity is seemingly controversial. On the one hand, genistein has been demonstrated to enhance SIRT1 expression and activity in C2C12 myotubes [23] and breast cancer T47D cells but not in MCF-7cells [33]. Moreover, genistein was found to increase AMPK (adenosine monophosphate activated protein kinase) activity in liver cells, which could trigger SIRT1 activity [34]. On the other hand, genistein has also been demonstrated to inhibit the expression of SIRT1 in prostate cancer cells [35]. A reasonable explanation for these phenomena is that genistein might have dual effects on SIRT1 expression and activity, and the effects depend on the different tissue contexts and dosage. Recently, one reporter provided another explanation for the phenomena. The modulation of the intrinsic gene expression by genistein could lead to a difference in radio-sensitivity in between cancerous and normal cells [36]. Interestingly, our study clearly demonstrated that the expression of SIRT1 was significantly increased 24 h after renal I/R injury following pretreatment with genistein (10 mg/kg and 15 mg/kg), and the inhibition of SIRT1 activity or expression abolished the protective effects of genistein on I/R-induced renal injury. Genistein treatment significantly reduced apoptosis and enhanced cellular proliferation rate, further supporting the protective effect of SIRT1. However, the inhibition of SIRT1 activity or its expression reduced the proliferation rate mediated by genistein. Moreover, a significant reduction in the p21 expression level occurred in genistein-treated mice. p21 is a cyclin-dependent kinase (CDK) inhibitor, and it might bind to and inhibit the activity of cyclin-CDK2 or cyclin-CDK4 complexes, thereby regulating cell cycle progression. Therefore, our study showed that genistein may exert its renal protective effects on renal I/R injury through its stimulation of SIRT1 expression or activity. SIRT1 expression or activity could be regulated directly or indirectly in vitro and in vivo. Hence, it is necessary to explore the definite mechanism regarding how SIRT1 expression and activity are regulated by genistein in renal cells.

**Figure 8:**
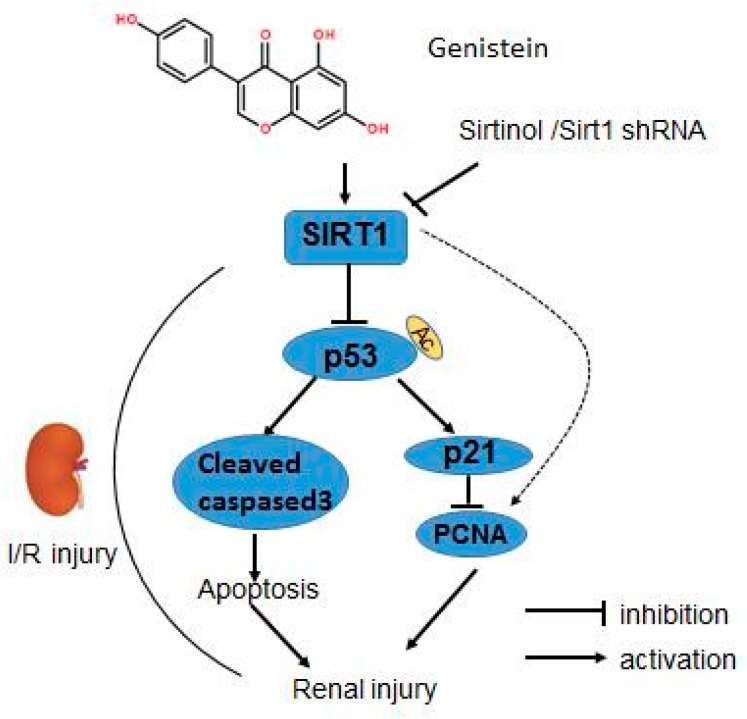
Proposed scheme for the roles of SIRT1, p53, and other molecules in the protective effect of genistein on I/R-induced renal injury. Genistein treatment significantly reduced renal I/R-induced cell death, simultaneously stimulating renal cell proliferation. Paralleling the protective effect of genistein against I/R-induced renal injury, SIRT1 expression was upregulated upon the administration of genistein. Genistein reduced p53, p21, and cleaved caspase-3 expression and increased PCNA expression. Pharmacological inhibition or shRNA-mediated depletion of SIRT1 significantly reversed the effect of genistein on renal dysfunction, cellular damage, apoptosis, and proliferation following I/R injury. The reduced p53, p21 expression, and increased PCNA expression were blunted after the depletion of SIRT1 compared with the genistein treatment group in the renal I/R process.

As a tumor suppressor, p53 is involved in multiple essential cell functions, such as pausing the cell cycle, promoting senescence and apoptosis, and regulating cell metabolism. The participation of p53 has been reported in nephrotoxic injury and ischemic renal injury, and targeted deletion of p53 in the proximal tubule prevents ischemic renal injury. p53 expression is negatively associated with SIRT1 expression and activity after AKI [14]. Kidney-specific overexpression of SIRT1 protects against AKI [30]. Furthermore, genistein decreases cisplatin-induced renal injury by preventing p53 induction [37]. Those data indicate that p53 expression is closely related to SIRT1 expression or activity after AKI. To test the potential relationships between genistein, p53, and SIRT1, we examined the level of p53, acetylated p53, p21, and PCNA (a proliferation marker) [13], respectively. Our study demonstrated that p53 and p21 were significantly decreased 24 h after renal I/R injury, accompanied by the induction of SIRT1 following pretreatment with genistein. The pivotal role of SIRT1 was further supported by the finding that the inhibition of SIRT1 activity or expression significantly blocks the repression of p53 expression, and p21 following the use of genistein. Moreover, there was the inverse correlation between p53, p21, and PCNA expression. It has shown that enhanced SIRT1 activity can reduce p53 expression by deacetylating p53 and promoting its ubiquitination and proteasomal degradation [38]. Genistein caused a robust reduction in p53 abundance but had no measurable effect on p53 acetylation associated with upregulated SIRT1 expression. It is plausible that SIRT1 caused the deacetylation of p53, contributing to p53 degradation by genistein. Remarkably, many factors contribute to the stability of p53 expression. These findings suggest the existence of other SIRT1-dependent mechanisms that tune p53 expression by genistein. Together, these data indicated that the SIRT1/p53 axis plays an important role in the protective effects of genistein on I/R-induced renal injury. In addition to regulation of DNA synthesis, PCNA has far-reaching impacts on a myriad of cellular functions by interaction with many protein and various post-translational modifications [39]. The protective roles of genistein in I/R-induced renal injury are associated with a higher PCNA level, implying the cellular proliferation involved at least in part in the protective effects of genistein. Moreover, it has to make great efforts to explore the more enigmatic PCNA-dependent function in this process, including senescence and apoptosis. In this study, we found that genistein could protect against I/R-induced renal injury, and the reno-protective effects of genistein were associated with reduced apoptosis and a higher renal proliferation rate. Surprisingly, the upregulation of SIRT1 expression is closely correlated with improved function against kidney injury in genistein-receiving mice. Pharmacological inhibition of SIRT1 abolished the reno-protective effects of genistein, and knockdown of SIRT1 abrogated the beneficial effects of genistein on kidney injury following I/R. Additionally, increased SIRT1 lead to reduced expression of p53, reduced apoptosis, and a higher renal cellular proliferation rate and vice versa, suggesting genistein may promote cell survival and enhance renal cellular proliferation via SIRT1/p53 axis to provide reno-protection in I/R-induced renal injury (Figure 8). The major isoflavones in soybean are genistein and daidzein. Daidzein can behave similarly to genistein and both of them have been shown to protect from metabolic disease in a SIRT1-dependent manner [40]. Daidzein has been shown to induce the expression and activity of SIRT1 in renal cells [40]. In the light of the protective effects of SIRT1 in kidney, daidzein administration could ameliorate I/R-induced kidney injury by stimulating the expression and activity of SIRT1. This needs to be testified in future study.

## 5. Conclusions

In summary, we confirmed that genistein possesses protective effects on renal ischemia-reperfusion injury and that the protective effect mainly depends on apoptosis inhibition and regeneration promotion accompanied by the increased expression of SIRT1, whereas inhibition of SIRT1 activity or expression blocked the protective effect. It is probable that genistein improves I/R-induced renal-injury in a SIRT1-dependent manner, making it a potential reagent for treatment of kidney disease with deficiency of SIRT1 activity. This need to be further verified using more in vivo and in vitro experiments.

## Appendix A

### Appendix A.1. Lentivirus Preparation

Lentiviruses were generated by cotransfecting subcofluent HEK293T cells with the lentiviral vectors (psh-control or psh-SIRT1) and packaging plasmids (pHR and pCMV-VSV-G) by calcium phosphate transfection. The targeted sequences for psh-SIRT1 and psh-control were 5′-AAGATGAAGTTGACCTCCTCA-3′ and 5′-TTCTCCGAACGTGTCACGT-3′, respectively. Viral supernatants were collected 48 h after transfection, centrifuged at 3000× *g* for 15 min, and filtered through 0.45-μm filters (Millipore, Danvers, MA, USA). The concentrations of lentivirus were determined using ultracentrifugation approaches [41].

### Appendix A.2. Quantitative Real-Time RT-PCR

Total RNA was extracted using TRIZOL Reagent (Invitrogen, Carlsbad, CA, USA) and was reverse transcribed with ReverTra Ace (Toyobo, Dalian, China) to produce cDNA. Real-time PCR was performed using SYBR (Synergy Brands) Green-based detection in StepOnePlus™ (ABI, Redlands, CA, USA) according to the manufacturer’s instructions and using the following primer pairs: SIRT1 (NM_019812) (Forward: 5′-TGCTGGCCTAATAGACTTGCAA-3′, Reverse: 5′-CAGGAACTAGAGGACAAGACGTCA-3′); 18S rRNA (NR_003278) (Forward:5′-GGTCATAAGCTTGCGTTGATTAAG-3′, Reverse: 5′-CTACGGAAACCTTGTTACGACTTT-3′).
